# Study of an Ecological Cement-Based Composite with a Sustainable Raw Material, Sunflower Stalk Ash

**DOI:** 10.3390/ma14237177

**Published:** 2021-11-25

**Authors:** Adrian Alexandru Șerbănoiu, Cătălina Mihaela Grădinaru, Nicanor Cimpoeșu, Dumitru Filipeanu, Bogdan Vasile Șerbănoiu, Nelu Cristian Cherecheș

**Affiliations:** 1 Faculty of Civil Engineering and Building Services, “Gheorghe Asachi” Technical University of Iași, 700050 Iași, Romania; serbanoiu.adrian@tuiasi.ro (A.A.Ș.); filipeanu@yahoo.com (D.F.); chereches@tuiasi.ro (N.C.C.); 2 Faculty of Material Science and Engineering, “Gheorghe Asachi” Technical University of Iași, 700050 Iași, Romania; nicanorcimpoesu@gmail.com; 3 Faculty of Architecture “G.M. Cantacuzino”, “Gheorghe Asachi” Technical University of Iași, 700050 Iași, Romania; bogdan-vasile.serbanoiu@academic.tuiasi.ro

**Keywords:** agro-waste, eco-friendly material, sustainable building materials, ecological concrete

## Abstract

The use of plant ash as a sustainable cementitious material in concrete composition is a widely researched subject in the construction domain. A plant studied so far more for its thermal insulation properties, sunflower, was analyzed in this study with regard to its ash effects on the concrete composition. The present research aimed to analyze the effects of a 2.5%, 5%, 7.5%, 10%, 15%, 20%, or 30% volume replacement of cement by sunflower stalk ash (SA), a sustainable cementitious material, on the concrete compressive strength at 28 days and three months, the flexural and splitting tensile strengths, the resistance to repeated freeze–thaw cycles, and the resistance to chemical attack of hydrochloric acid. The elementary chemical composition of the SA and the composites was included also. According to the experimental results, SA decreased the values of the compressive and tensile strength of the concrete, but it improved the concrete behavior under repeated freeze–thaw cycles and under the action of hydrochloric acid. A percent of 10% of SA led to a much more pronounced development of compressive strength over time than conventional concrete (26.6% versus 12%).

## 1. Introduction

Sustainability became an important preoccupation of the twenty-first century in all industries, including the building materials industry. The building industry is the third largest CO_2_-emitting industry worldwide due to the levels recorded by Portland cement production. Yearly, 3.0–3.6 Gt of cement is produced, leading to around 3.24 billion tons of CO_2_ (5% of the total CO_2_ emissions) [1]. These kinds of numbers have determined perpetual research at the global level for finding solutions to diminish the high negative impact of this industry on the environment. The pathways for achieving this aim are numerous, varying from the use of more sustainable fuels from wastes for the burning process implied in obtaining Portland cement clinker [2] to identifying other cementitious materials, generically called supplementary cementitious materials (SCMs), with high ecologic and sustainable characteristics [3,4,5]. From SCMs category, agro-waste ash, among others, is often used, because its use for developing sustainable construction materials has the potential to achieve environmental, economic, and social sustainability in the long term [6].

The development of concrete compositions based on renewable raw materials is a measure that diminishes the negative effects of conventional concrete made with Portland cement on the environment. Regarding the use of plant ashes in cement-based composites, various studies have been conducted, a few examples being rice husk ash [7,8,9], sugarcane bagasse ash and palm oil fuel ash [9,10,11,12], wheat straw ash [13,14], and corn cob ash [15,16,17], among others.

A plant of interest for the building materials sector, widely spread across the world, is the sunflower, studied until now mostly as raw material for developing sustainable materials with insulation characteristics [18,19,20,21,22], due to its similar internal structure to hemp and polystyrene, with a very high content of air. Globally, sunflower is cultivated on around 26.03 × 10^6^ ha, with a yield of 2.07 tons/ha [23]. It represents an important crop, especially in southern South America, Southern Europe, South Africa, and South and European Russia [24]. Sunflower stalk is considered agricultural waste that remains on the cropland [25]. It does not have nutritional qualities for animal feed and it is not used as a material for heating and domestic cooking like other agricultural waste (wheat or corn). The sunflower leaves are used as fodder, the seeds for oil production, and the resulting oil cake after oil extraction for stock and poultry feeding [26].

Sunflower stalks can be a raw material for the construction industry, being an annually renewable resource, cheap, and widely available. To the best of our knowledge, in the scientific literature, there are very few studies on the use of ash from sunflower stalks to make cement-based composites. These studies have revealed that this sustainable raw material has pozzolanic qualities, leading to improved concrete durability and good resistance to sodium sulfate. Aksoğan et al. [14] studied the effects of sunflower stalk ash (SA) on concrete made with barite and colemanite. The optimum replacement rate found by the authors to have positive effects on compressive strength, abrasive resistance, and linear absorption coefficient was 2.5% of SA as a cement replacement. The mix with 5% of SA obtained increased resistance to 180 days of sulfate action in 5% sodium sulfate solution than the reference. SA also improved the concrete behavior subjected to freeze–thaw cycles [14]. Darweesh [26] studied the effects of SA on the physical, chemical, and mechanical properties of cement pastes, and found that SA can enhance the compressive strength, improves the CSH amount, and decrease the free lime content. The author concluded that the optimum rate of cement replacement with SA was 24 wt%, and higher rates such as 30 wt% negatively affect the properties of cement pastes.

Through this study, we wanted to make new concrete compositions with improved performance compared to those of classic concrete with Portland cement, but to be adapted to the new requirements related to energy efficiency in their production process or their durability. The aim of this research was to develop several variants of environmentally friendly concrete, by partially replacing cement with an easily renewable raw material, namely, sunflower stalk ash. Analyses performed on the developed cement-based compositions included the elementary chemical composition of SA and of the composites, mechanical tests (compressive strength at 28 days and three months, and flexural and splitting tensile strength) and durability tests for the action of repeated freeze–thaw cycles and the action of hydrochloric acid.

## 2. Materials and Methods

### 2.1. Materials

The analyzed compositions in this study were the following:

- A reference composition of microconcrete, RC, with cement, sand, and river gravel aggregates up to 8 mm in diameter; the water/cement (w/c) ratio used was 0.5.
- Seven cement-based composite mixes with SA as a partial replacement material for cement, in volume proportions of 2.5%, 5%, 7.5%, 10%, 15%, 20%, and 30%, the notations applied being CSA2.5, CSA5.0, CSA7.5, CSA10, CSA15, CSA20, and CSA30. The w/c ratio applied was increased every 10% step of used SA: In the CSA2.5, CSA5.0, CSA7.5, and CSA10 mixes, a 0.5 w/c ratio was used; in CSA15 and CSA20, a 0.518 w/c ratio was used; and in CSA30, a 0.536 w/c ratio was used. These w/c ratio differences were applied in order to maintain the same workability level for the fresh composite material.

The reference composition, RC, was realized by: Portland cement CEM II/A-LL42.5R type (HeidelbergCement Romania, Bucharest, Romania), 473 kg/m^3^; sand (sort 0–4 mm), 673 kg/m^3^; river gravel (sort 4–8 mm), 432 kg/m^3^; water, 2365 L superplasticizer additive based on polycarboxylateter, 2% of binder.

The notations used in the cement label denote the following characteristics:

▪ CEM II: Portland-composite cement.
▪ A-LL: Portland limestone cement that contains 80–94% clinker of the total mass, 6–20% limestone, and 0–5% additional constituents; limestone consists of a maximum of 75% calcium carbonate (CaCO_3_) of the total mass of calcium oxides, clay a maximum of 1.20 g/100 g of limestone, and a maximum of 0.20% of total organic carbon of the total mass of limestone.
▪ 42.5R: Belongs to the standard 42.5 strength class with high early strength; the compressive strength developed by this cement is greater than or equal to 20 MPa in two days, and between 42.5 and 62.5 MPa in 28 days, with an initial setting time greater than or equal to 60 min and with an expansion smaller than 10 mm.

### 2.2. Methods

#### 2.2.1. Sunflower Stalk Ash Preparation

The method adopted to obtain the SA was free burning in a refractory brick kiln. Bie et al. (2015) [7] studied the influence of burning temperature on rice husk, and found that at 600 °C, the amorphous form of rice husk ash can be obtained. Chindaprasirt et al. (2008) [9] stated that a 650 °C burning temperature is optimum to achieve higher pozzolanic activity. At these temperatures, the crystallization phenomenon does not take place and the amorphous form of the ash, with an important role in pozzolanic activity, is preserved. The method applied in our study followed these details and tried to achieve these temperatures by free burning. The result was, instead, a 700 °C temperature. This is the temperature found by Riberio et al. (2014) [12] to be optimum to achieve better mechanical properties of a mortar made with a 10% replacement rate of cement with sugarcane bagasse ash.

**Figure 1:**
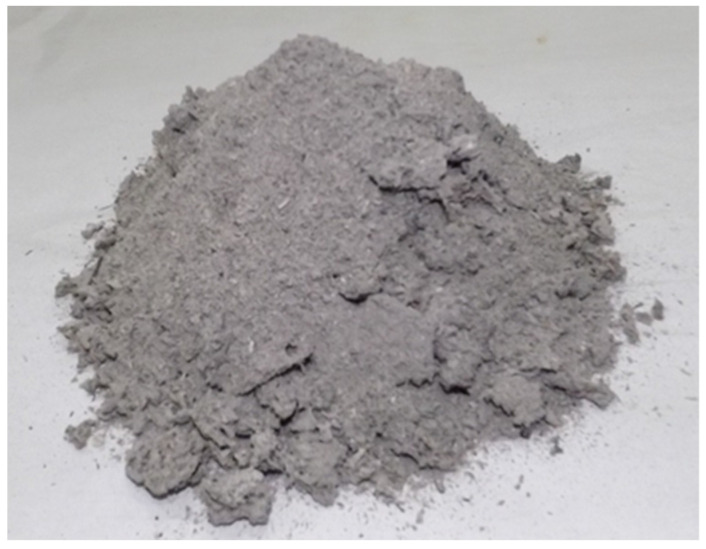
Raw ash resulted from the free burning of sunflower stalks.

**Figure 2:**
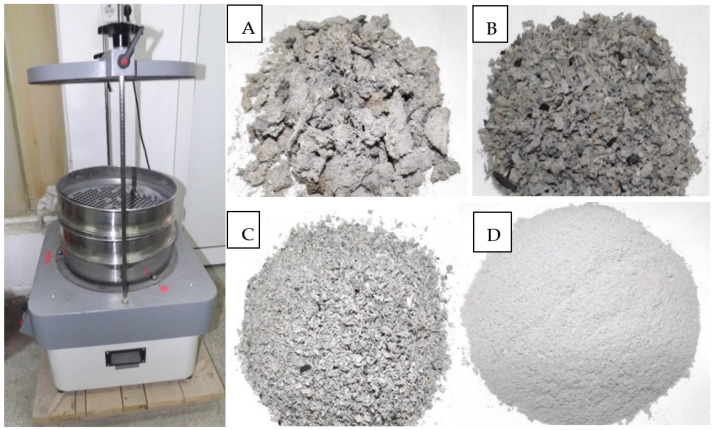
The sieving equipment and steps for sieving ash from sunflower stalks obtained by free burning: (**A**) Material left in the 20 mm sieve after 5 min of sieving; (**B**) material left in the 10 mm sieve after 5 min of sieving; (**C**) material left in the 2 mm sieve after 5 min of sieving; (**D**) material passed through the 300 µm (0.3 mm) sieve after 5 min of sieving.

According to Bahuradeen et al. (2015) [11], the pozzolanic activity of burnt ash is better if its particles are smaller than 300 µm. So, the raw ash obtained by free burning (Figure 1) in our study was sifted for 5 min through multiple sieves (20 mm, 10 mm, and 2 mm) up to 300 µm, using automatic sieving equipment (Endecotts Powermatic Test Sieve Shaker) (Figure 2).

**Figure 3:**
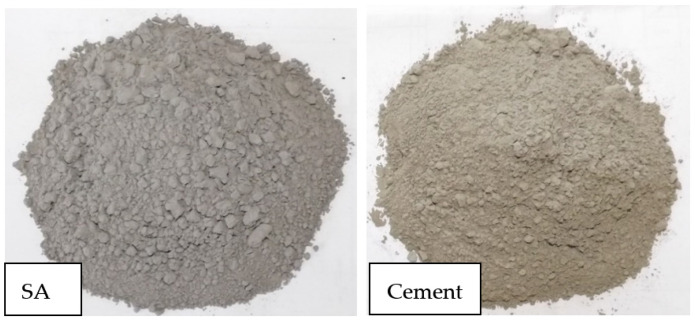
Sunflower ash resulted from ball grinding for 120 min. and used in mixes, compared to the cement.

The ash that passed through the 300 µm (0.3 mm) sieve after 5 min of sieving was subjected to a grinding process for 120 min in a ball dust crusher. The obtained sunflower ash (SA) (Figure 3) was used as the cement replacement in our study. It can be observed in Figure 3 that the SA has a greater gray tint than the cement due to its carbon content.

#### 2.2.2. Sunflower Stalk Ash Analysis

The composition and aspect of SA was analyzed using scanning electron microscopy (SEM; VegaTescan LMH II, SE detector, 30 kV, Tescan Orsay Holding, Brno–Kohoutovice, Czech Republic) coupled with an energy dispersive X-ray spectrometer detector (EDS; Bruker XFlash 6I30, automatic mode, Bruker, Billerica, MA, USA).

#### 2.2.3. Composite Mix Preparation

In this research, cement-based composites with 0%, 2.5%, 5.0%, 7.5%, 10%, 15%, 20%, and 30% sunflower stalk ash as a cement substitute were developed. The mixes were made according to NE 012/1-2007 [27]. For the preparation of these mixes, a portable electric concrete mixer was used. After 24 h from pouring into molds, the concrete specimens were unmolded and left to cure in ambient conditions for 28 days. The ambient conditions were a 20 ± 3 °C air temperature and a 55 ± 10% relative humidity.

#### 2.2.4. Composite Specimen Properties

##### Composition Analysis

The composition and aspect of the specimens were analyzed using scanning electron microscopy (SEM; VegaTescan LMH II, SE detector, 30 kV, Tescan Orsay Holding, Brno—Kohoutovice, Czech Republic) coupled with an energy dispersive X-ray spectrometer detector (EDS; Bruker XFlash 6I30, automatic mode, Bruker, Billerica, MA, USA). A low-vacuum mode was necessary to analyze the samples based on the fact that all of the samples gassed quite hard. Cubes samples (1 cm^3^ volume) were mechanically cut and prepared for analyses.

##### Mechanical and Durability Property Analysis

In Table 1 are presented, in short, the essential elements for the followed methods in the performed tests regarding compressive strength, flexural tensile strength, splitting tensile strength, resistance to freeze–thaw, and resistance to chemical attack of hydrochloric acid action.

In order to determine the characteristics of cement-based composites, test specimens were made by casting in metal molds in the form of cylinders with a diameter of 100 mm and a length of 200 mm, prisms measuring 100 × 100 × 550 mm^3^, and cubes measuring 100 × 100 × 100 mm^3^ and 50 × 50 × 50 mm^3^. The cylinders were used to determine the compressive strength and splitting tensile strength of the composites, the prisms to determine the flexural tensile strength, the cubes of a 100 × 100 × 100 mm^3^ dimension to determine the resistance after 50 freeze–thaw cycles, and cubes of 50 × 50 × 50 mm^3^ to determine the resistance to the action of hydrochloric acid. The tests were performed at 28 days. In the case of mechanical tests, three specimens were tested for each mixture and each test, according to the standards in force, and the average value was taken into account in the analysis tests. The freeze–thaw resistance test involved the testing of six specimens for each mix, as three specimens had the role of control samples, not subjected to the freeze–thaw process, and three specimens were subjected to the action of 50 freeze–thaw cycles.

The mechanical tests were performed with a hydraulic press of 60 kN for performing tests on concrete.

##### Resistance to Repeated Freeze–Thaw Cycles

The cube specimens with sides of 100 mm were subjected to 50 freeze–thaw cycles, according to the SR 3518:2009 [31] stipulations. Before the effective test started, six specimens for each mix were immersed gradually in water of 20 ± 5 °C in temperature, first up to ¼ of their height for 24 h, then up to ½ of their height for next 24 h, then up to ¾ for another 24 h, and, finally, totally immersed for another 24 h. After the immersion process, three specimens for each mix remained in the water bath, while the other three were subjected to 50 freeze–thaw cycles. A freeze–thaw cycle lasted 8 h: During the first 4 h, the specimens were frozen in a cold room that maintained the temperature at −17 ± 2 °C; then, in the next 4 h, the specimens were introduced to water of 20 ± 5 °C in temperature. After the 50 cycles, all of the specimens, including those kept in water all the time, were tested for compressive strength, according to EN 12390-3:2019 [28], and the freeze–thaw resistance evaluation was made as the difference between the average value of the three specimens always kept in water and the average value of the three specimens subjected to the freeze–thaw process.

##### Resistance to Chemical Attack of Hydrochloric Acid (HCL)

**Figure 4:**
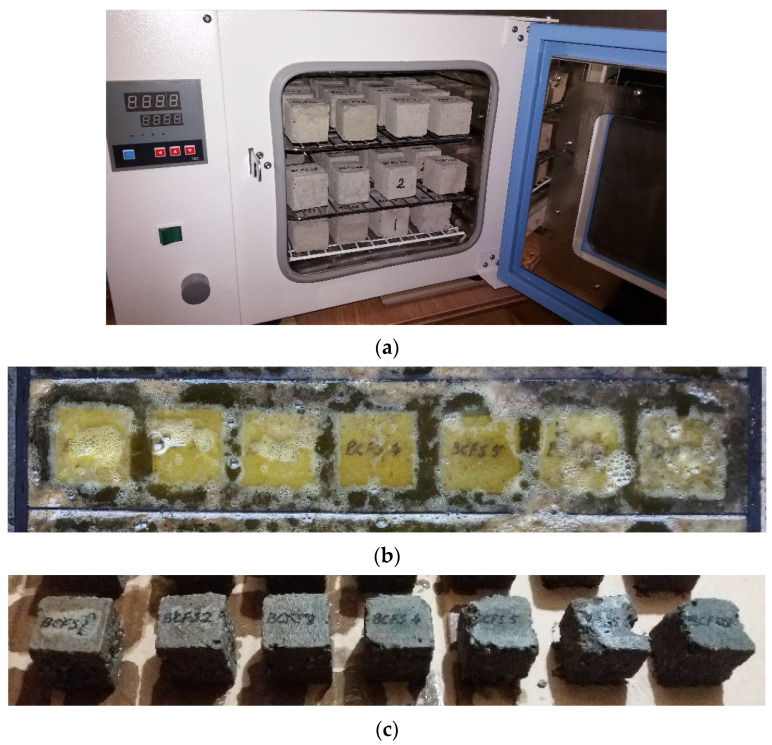
Resistance to the action of hydrochloric acid. (**a**) The specimens drying in an oven; (**b**) The specimens immersed in a 18% HCl solution; (**c**) The specimens after 10 days of immersion in the 18% HCl solution.

To test the resistance to chemical attack of hydrochloric acid (Figure 4), three cubes with sides of 50 mm were used for each mix. They were first dried in an oven (Figure 4a) at 90 °C until constant mass and then weighed. Subsequently, they were immersed in a HCl solution of an 18% concentration (Figure 4b) for 10 days. This high concentration was adopted in order to shorten the test period and to subject the compounds to the most aggressive action of this substance. After 10 days, the specimens were washed with clean water, brushed to remove all particles that came off the body of the cubes (Figure 4c), then placed in the oven at 90 °C and dried up to constant mass, and finally weighed. The resistance assessment consisted of determining the average relative mass loss.

## 3. Results and Discussion

### 3.1. Sunflower Stalk Ash and Cement

According to quantitative EDS analysis (Table 2), SA presented the following elementary chemical composition, expressed in mass percentages (wt%): Oxygen—50.81%, potassium—22.85%, calcium—10.18%, carbon—7.91%, magnesium—5.41%, chlorine—2.16%, and silicon—0.68%. Comparative to the cement, SA did not contain aluminum, iron, or sulfur. The other important differences were the high level of potassium, the very small quantity of silicon, and the presence of carbon, magnesium, and chlorine. The high content of C and K had a strong influence on the strength of the mixes, as well as the small content of Si.

**Figure 5:**
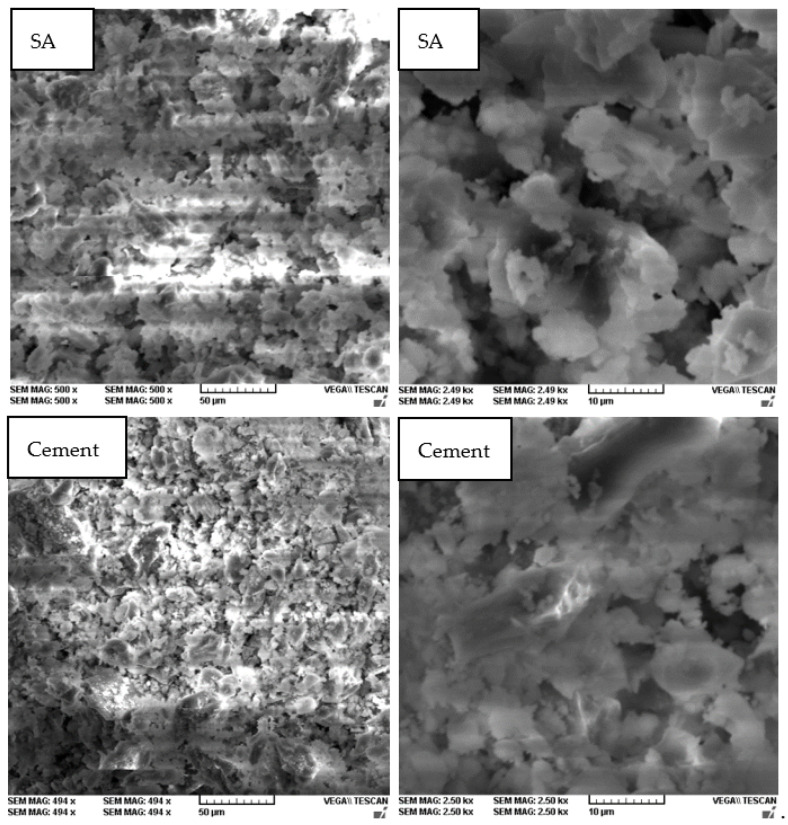
SEM images of the SA and cement at a magnification rate of 50 µm (**left** images) and of 10 µm (**right** images).

Scanning electron microscopy (SEM; Figure 5) presented a porous structure, characteristic of these type of materials. According to the SEM images, the structure of the SA was more compact than that of the cement, forming bigger granule agglomerations. However, the more compacted structure involved bigger air voids than the cement.

**Figure 6:**
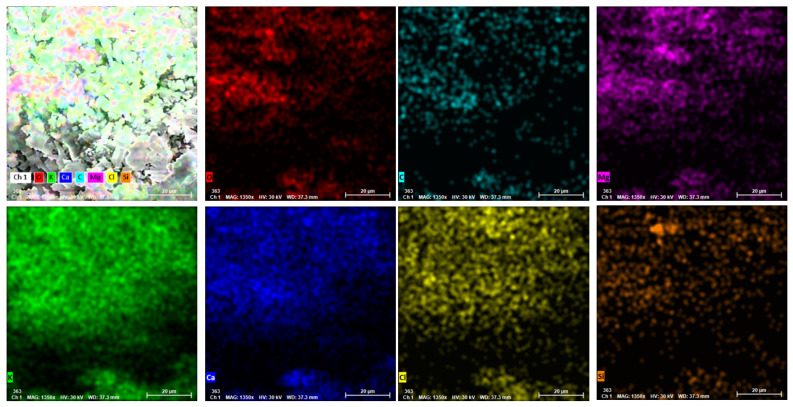
SEM images of the chemical elements of SA at a magnification rate of 20 µm.

In Figure 6 are presented the distributions of the main identified elements on a 0.01 mm^2^ area: O, C, Mg, K, and Ca. Beside the general oxides mass, few small compounds based on Si or Cl could be observed.

### 3.2. Composite Specimen Properties

#### 3.2.1. Chemical Composition Analysis

In Table 3, the chemical composition of the studied cement-based composites, according to EDS analysis, is presented from the mass and atomic percentages point of view.

From the identified elements in the composites structure, Si and Ca have important roles in forming calcium–silicate–hidrate (CSH), which leads to smaller permeability of the cement matrix and, consequently, to a better resistance to chemical attack [26]. The C, Al, and Fe contents influence the mechanical properties of concrete.

The Si content was almost the same in the RC and CSA2.5 (20.8 ± 0.1 wt%), and slightly increased in CSA7.5 (22.12 wt%), CSA5 (24.86 wt%), CSA10 (25.22 wt%). CSA15 and CSA20 presented the highest Si content of all of the mixes: 28.48 wt% and 33.66 wt%, respectively. CSA30 had a much smaller Si content of 13.73 wt%.

The C content was smaller in all CSA mixes, comparative to RC, although it was missing in CSA20 and was higher in CSA30, with 2.73 wt%, compared to RC.

The Ca content was quite similar in CSA7.5 and RC, namely, 10.4 wt%. In CSA2.5 and CSA30, it was higher: 12.13 wt% and 16.32 wt%, respectively. In the other CSA mixes, it decreased up to 5.44 wt%.

Al was present in the highest amount in CSA5: 4.04 wt%. In CSA10 and CSA15, the Al content decreased to 2.6–2.7 wt%, and in CSA7.5 and CSA20 to around 2 wt%. The smallest quantities of Al were found in the RC and CSA30: Around 1.1%.

Regarding the Fe content, the highest level was found in CSA20 and CSA5, at 2.39 wt% and 2.02 wt%, respectively. The other mixes had smaller Fe contents, with around 50%.

Na and K elements were present in small quantities (between 0.7 and 1.4 wt%) and only in a few mixes. Mg was found only in CSA30, in a very small rate of around 1 wt%.

**Figure 7:**
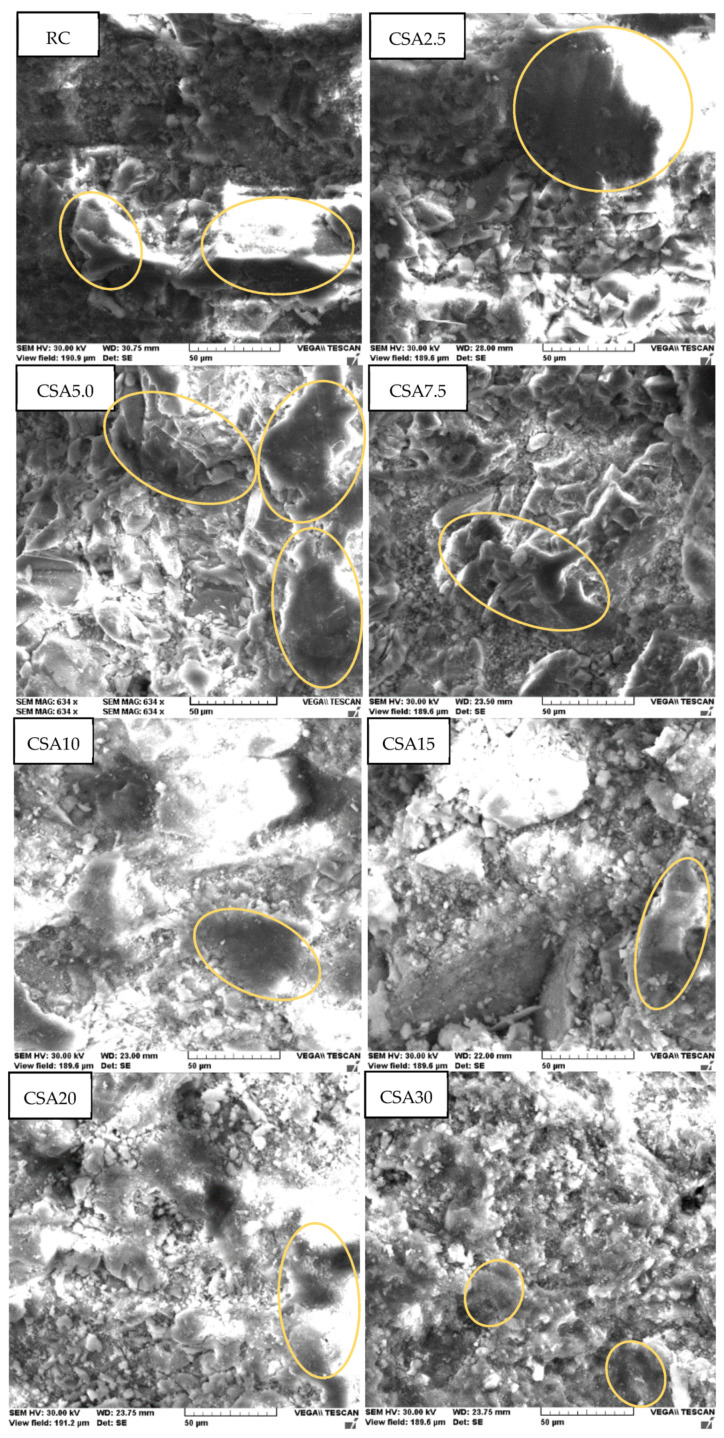
SEM images at a 50 µm magnification rate of the studied cement-based composites.

In Figure 7 are presented the SEM images of the developed mixes. It can be observed that internal structure became less compact (lower CSH content—exemplified by some areas highlighted by encirclement) as the SA rate increased.

**Figure 8:**
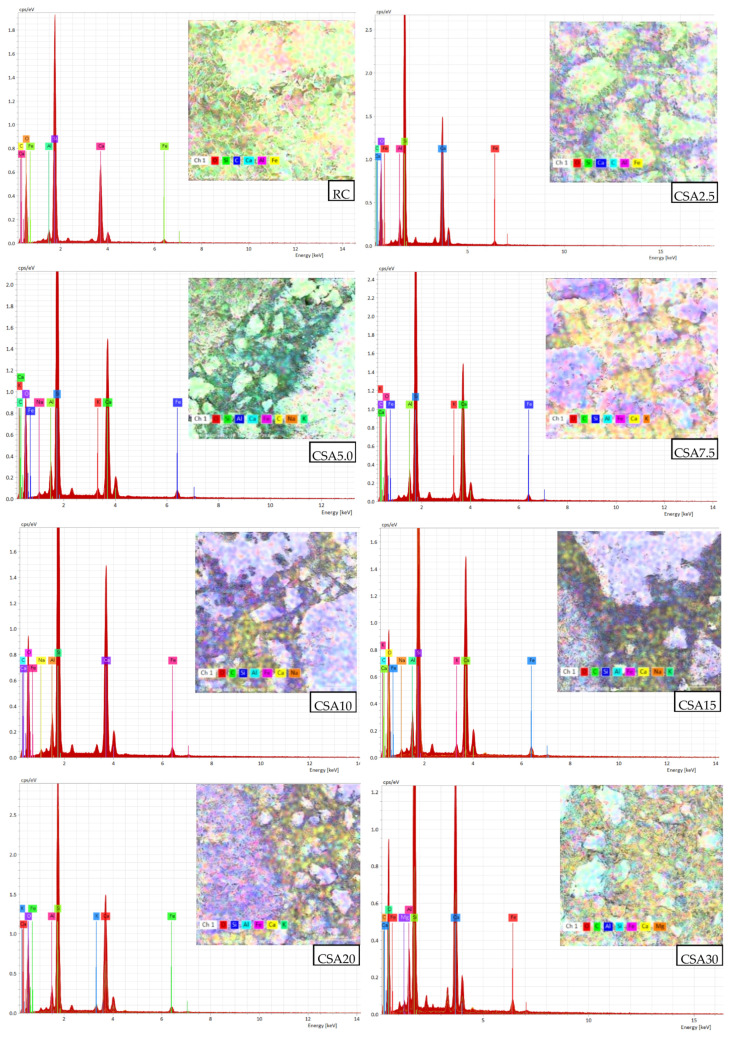
EDS analysis and SEM images at a 400 µm magnification rate of the studied cement-based composites.

In Figure 8 are presented the chemical elements of the studied mixes.

#### 3.2.2. Compressive Strength

**Figure 9:**
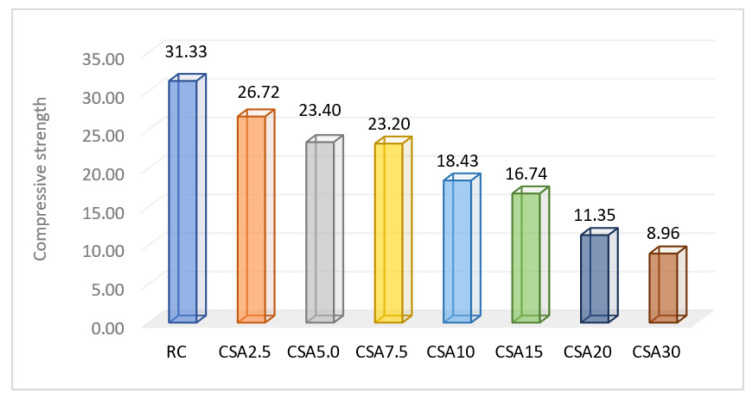
Compressive strength of the cement-based composites at 28 days (N/mm^2^).

The compressive strength determined at 28 days is presented in Figure 9. It can be observed that SA led to smaller compressive strengths. In CSA2.5, CSA5.0, CSA7.5, and CSA10, an equal step of cement replacement with SA from one mix to another was not maintained in terms of the evolution of the compressive strength values obtained by them. The first 2.5% replacement rate led to a 14.71% decrease compared to RC. Then, CSA5.0 and CSA7.5 both registered almost the same compressive strength—23.40% and 23.20%, respectively—smaller by around 12.80% then CSA2.5. CSA10 had a bigger decrease in this parameter than the anterior variant, CSA7.5, with 20.56%. The same situation of an unequal effect was also observed in the cases of CSA10, CSA20, and CSA30. The replacement rate increased by 10%, but this did not have the effect of evenly decreasing the compressive strength from one mix to another. The compressive strength of CSA20 decreased by 38.41% compared to CSA10, and CSA30 by 21.06% compared to CSA20. CSA20 registered a smaller value with 60% more than RC, and CSA30 with around 70% less.

If we take into account the optimum rate between the cement quantity replaced and the compressive strength value, the best result was obtained by CSA7.5 regarding the compressive strength at 28 days.

**Figure 10:**
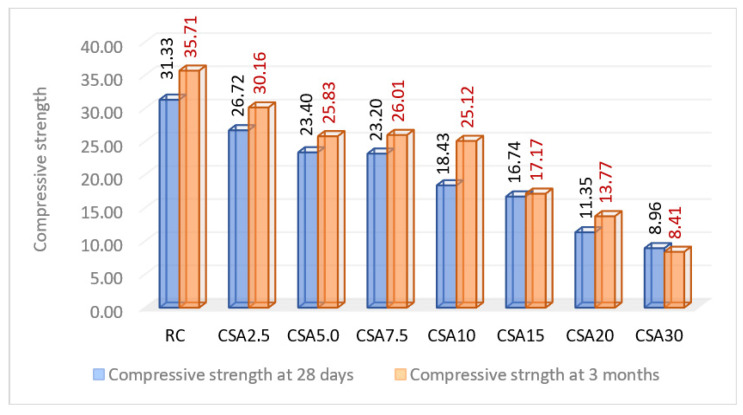
Compressive strength variation at the 28 days and three months of age (N/mm^2^).

In this research, the evolution of the compressive strength over time was studied, at three months after casting (Figure 10), given the well-known fact that this parameter records increases in the measured values at various time intervals after the initial determination. As evidence, according to Figure 10, it can be seen that the RC recorded an improvement in compressive strength by approximately 12%. Analyzing the results of the compositions with vegetal ash, CSA10 and CSA20 obtained higher values than RC, registering an increase in this strength of approximately 27% and, respectively, 18% compared to the value measured at 28 days. CSA2.5, CSA5.0, and CSA7.5 recorded relative increases close to those of RC, but below its level. In the case of the composition of CSA30, the high percentage of vegetal ash caused a slight decrease in the compressive strength over time. As regards the compressive strength at three months, CSA10 had the best evolution among all of the studied compositions (Figure 10), in the sense that, over time, there was a decrease in the gap between its compressive strength and that of RC; at 28 days, this gap was approximately 41%, and at three months, it was approximately 30%. In the case of the other compositions, the RC versus CSA compressive strength gap did not undergo major changes.

#### 3.2.3. Flexural Tensile Strength

**Figure 11:**
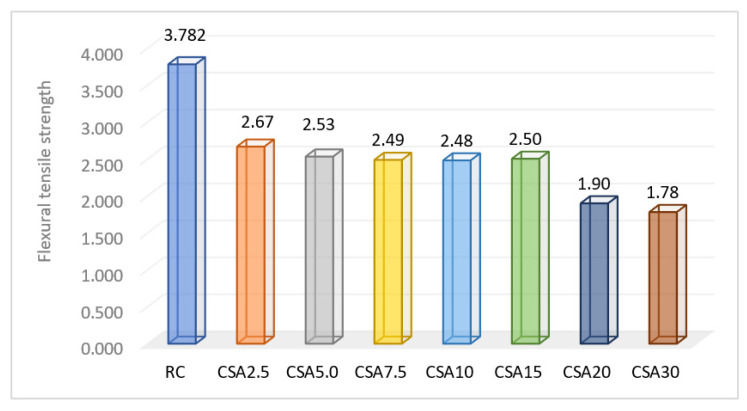
Flexural tensile strength of the composite samples (N/mm^2^).

The SA used in the concrete composition led to a decrease in the flexural tensile strength (Figure 11). The replacement rates of the cement between 2.5% and 15% determined a decrease in this parameter to approximately the same extent, with a rate of around 31 ± 2%. Thus, CSA2.5 registered a decrease of 29.5% of this parameter, and CSA5.0, CSA7.5, CSA10, and CSA15 obtained values close to one another, by approximately 35.5 ± 0.5% higher than RC. The 20% and 30% of SA led to around a 50% decrease compared to RC.

The best results in terms of the flexural tensile strength were obtained by CSA15, if the aim is to use as much SA ash as possible.

#### 3.2.4. Splitting Tensile Strength

**Figure 12:**
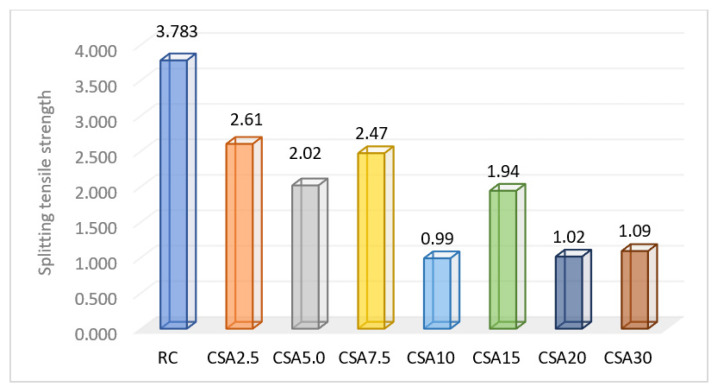
Splitting tensile strength of the composite samples (N/mm^2^).

The SA determined decreases in the case of splitting tensile strength as well, but its influence in the concrete composition was not a predictable one (Figure 12). The obtained results revealed that applying a replacement rate of 7.5% had almost the same effect as that of 2.5%, namely a decrease of 33 ± 1.5% than RC. Moreover, the 5% and 15% rates determined similar diminishes of around 47 ± 1%. The other three studied replacement rates—10%, 20%, and 30%—decreased this parameter value by approximately 72.5 ± 1%.

The composition that can be considered optimal in terms of the splitting tensile strength, taking into account the highest possible replacement rate, is CSA7.5.

#### 3.2.5. Resistance of the Composites to Repeated Freeze–Thaw Cycles

**Figure 13:**
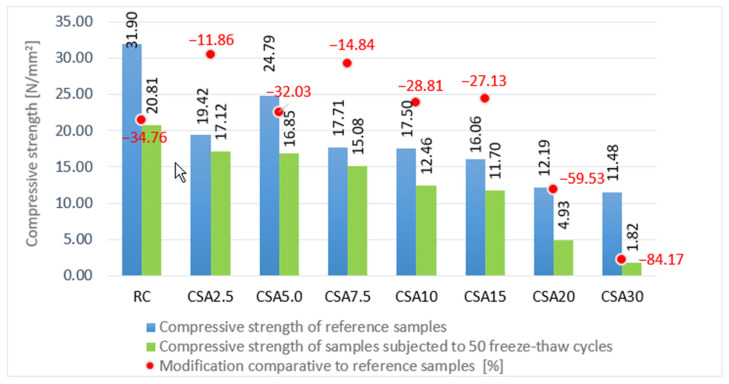
Variation in the compressive strength of the samples after testing for 50 freeze–thaw cycles, comparative to the non-frozen samples (%).

The SA positively influenced the concrete resistance to freeze–thaw when up to 15% of the cement volume was used (Figure 13). After sample testing of 50 freeze–thaw cycles, the compressive strength of the RC decreased by around 35%. Partial replacement of cement with SA had positive effects up to the 15% level. Thus, a small rate of 2.5% of SA led to a decrease of only 12%, while for the 7.5% rate, the decrease was around 15%. The 15%, 10%, and 5% rates led to a decrease that was also smaller than that of the RC, namely, by around 27%, 29%, and 32%, respectively. Instead, the 20% and 30% rates determined a much smaller resistance of concrete to freeze–thaw—decreases of around 60% and 84%, respectively, being obtained, compared to the control samples, for those not subjected to the freeze–thaw process.

In terms of the best ratio of freeze–thaw resistance versus percentage of SA used, CSA7.5 can be considered the optimal composition.

#### 3.2.6. Resistance to Chemical Attack of HCl

**Figure 14:**
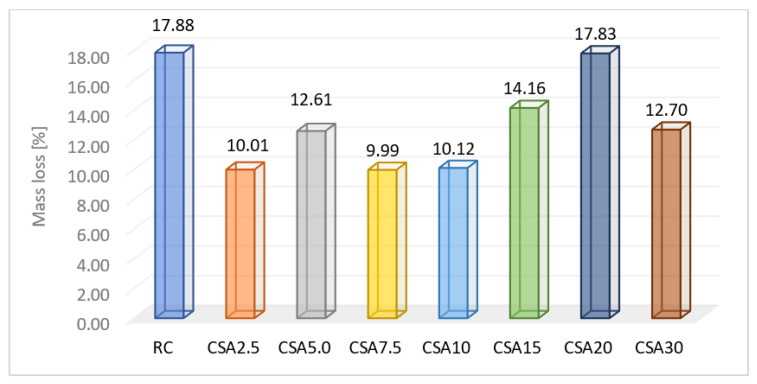
Mass loss due to the action of the 18% HCl solution action (%).

Following the action of the 18% HCl solution (Figure 14), it could be observed that the SA had positive effects in terms of resistance of the studied compositions. The analysis of the obtained results was performed based on the comparison of the relative mass loss of the samples following the test. Thus, it could be observed that the RC registered a mass loss of 17.88%. Compared to the RC, the best results were obtained by CSA2.5, CSA7.5, and CSA10, for which relatively similar weight losses of approximately 10% were measured, which were around 44% smaller than that of the RC. CSA5.0 and CSA30 recorded losses of approximately 13%, CSA15 of 14%, and CSA20 of around 18%. Compared to RC, applying the 5% and 30% replacement rates led to an improvement of around 29%, and the 15% rate to improvements of around 21%. CSA20 registered, instead, almost the same resistance as the RC.

The composition that can be considered optimal in terms of durability to the action of hydrochloric acid versus the cement replacement rate is CSA10.

## 4. Conclusions

The research consisted of the analysis of the influence of sunflower stalk ash as a substitute for 2.5%, 5%, 7.5%, 10%, 15%, 20%, and 30% of the cement volume in the composition of a microconcrete on the compressive strength, flexural and splitting tensile strengths, freeze–thaw resistance, and chemical resistance to a hydrochloric acid solution.

As a partial replacement of cement, SA led to a decrease in the mechanical performance of concrete, and the obtained results did not follow a predictable logic, in accordance with the replacement step applied. On the contrary, SA determined the improvement of the concrete resistance to freeze–thaw.

As chemical composition, CSA mixes contain, in general, supplementary chemical elements compared to RC, with the presence of potassium and magnesium being assumed to be due to the SA composition. The chlorine found in the SA composition was not detected in any of the CSA mixes.

Taking into account the results obtained in all of the tests performed in this study, CSA7.5 seems to be the optimum mix.

As a result, this sustainable raw material, SA, could be part of the concrete used to make prefabricated elements used outdoors, such as pavements, curbs, drains, gutters, irrigation systems (manholes, pipes, and canals), and the like. The realization of such elements with increased durability would lead to a decrease in their replacement rate due to cyclical deterioration of natural causes; therefore, it would also lead to obtaining economic gains over time. If we take into account the quantities used to make pavements for pedestrian alleys, for example, and their replacement rate, we can largely predict the economic benefit obtained, and the use of bio-based construction composites with SA could be a solution to be considered in the future.

To achieve improved resistance to the attack of hydrochloric acid, it is recommended to use SA as a raw material in the composition of concrete to be used in the construction of buildings intended for production activities of dyes, organic fertilizers, galvanized sheet, leather processing, etc., as these activities involve the use of hydrochloric acid; therefore, these buildings must be protected from the action of this substance.

Given that SA has reduced mechanical strength but improves durability, it is considered that research should be continued in terms of using SA as an sustainable additive in the composition of cement-based composites and not as a substitute for cement, in order to obtain a material with improved performance in terms of mechanical characteristics as well, not just as durability.

## Figures and Tables

**Table 1:** Specifications for the mechanical and durability tests.

Test	Specimen Type and Dimensions	Specimen Dimensions	Specimen Number Tested for Each Mix	Standard Applied
Compressive strength	Cylinder	100 mm diameter 200 mm length	3	EN 12390-3:2019 [28]
Flexural tensile strength	Prism	100 × 100 mm^2^ transversal section 550 mm length	3	EN 12390-5:2019 [29]
Splitting tensile strength	Cylinder	100 mm diameter 200 mm length	3	EN 12390-6: 2010 [30]
Resistance to freeze–thaw	Cube	Sides of 100 mm	6	SR 3518: 2009 [31] EN 12390-3:2019 [28]
Resistance to hydrochloric acid action	Cube	Sides of 50 mm	3	Figure 4

**Table 2:** EDS analysis of the SA and cement: Quantitative identification.

Element	SA		Cement	
	Mass Norm. [%]	Atom [%]	Mass Norm. [%]	Atom [%]
Oxygen (O)	50.81	63.76	47.65	64.17
Aluminum (Al)	-	-	12.89	10.29
Potassium (K)	22.85	11.73	1.07	0.59
Calcium (Ca)	10.18	5.10	12.12	6.51
Carbon (C)	7.91	13.23	-	-
Magnesium (Mg)	5.41	4.47	-	-
Chlorine (Cl)	2.16	1.22	-	-
Silicon (Si)	0.68	0.49	20.97	16.09
Iron (Fe)	-	-	4.25	1.64
Sulfur (S)	-	-	1.05	0.70
SUM	100	100	100	100

**Table 3:** Chemical composition of the studied cement-based composites, according to the EDS analysis.

Element	RC		CSA2.5		CSA5.0		CSA7.5		CSA10		CSA15		CSA20		CSA30	
	Mass [%]	Atom [%]	Mass [%]	Atom [%]	Mass [%]	Atom [%]	Mass [%]	Atom [%]	Mass [%]	Atom [%]	Mass [%]	Atom [%]	Mass [%]	Atom [%]	Mass [%]	Atom [%]
Oxygen	53.96	*61.22*	52.34	*60.75*	51.54	*62.22*	53.22	*62.49*	52.10	*61.45*	51.69	*61.06*	53.87	*68.94*	51.15	*58.09*
Silicon	20.69	*13.38*	20.94	*13.84*	24.86	*17.10*	22.12	*14.80*	25.22	*16.95*	28.48	*19.17*	33.66	*25.54*	13.73	*8.88*
Carbon	13.02	*19.68*	11.80	*18.24*	7.22	*11.61*	9.90	*15.48*	9.07	*14.25*	8.75	*13.77*	-	*-*	15.75	*23.83*
Calcium	10.39	*4.71*	12.13	*5.62*	7.74	*3.73*	10.45	*4.99*	8.43	*3.97*	5.44	*2.56*	7.04	*3.60*	16.32	*7.40*
Aluminum	1.13	*0.76*	1.75	*1.20*	4.04	*2.89*	2.12	*1.48*	2.64	*185*	2.70	*1.89*	1.96	*1.49*	1.15	*0.77*
Iron	0.81	*0.26*	1.04	*0.35*	2.02	*0.70*	1.29	*0.43*	1.13	*0.38*	1.16	*0.39*	2.39	*0.88*	0.93	*0.30*
Sodium	-	*-*	-	*-*	1.39	*1.17*	-	*-*	1.41	*1.15*	0.86	*0.71*	-	*-*	-	*-*
Potassium	-	*-*	-	*-*	1.19	*0.59*	0.70	*0.34*	-	*-*	0.91	*0.44*	1.08	*0.56*	-	*-*
Magnesium	-	*-*	-	*-*	-	*-*	-	*-*	-	*-*	-	*-*	-	*-*	0.97	*0.73*
SUM	100	*100*	100	*100*	100	*100*	100	*100*	100	*100*	100	*100*	100	*100*	100	*100*

## Data Availability

All data are contained within the article.
